# Widespread pulmonary invasion by malignant pleural mesothelioma: an important diagnostic consideration

**DOI:** 10.1002/rcr2.675

**Published:** 2020-10-26

**Authors:** Mia MacMillan, Bapti Roy, Sally McLaren, Anna K. Nowak, Rajesh Thomas, Y. C. Gary Lee

**Affiliations:** ^1^ Department of Respiratory Medicine Sir Charles Gairdner Hospital Perth WA Australia; ^2^ PathWest Laboratory Medicine Perth WA Australia; ^3^ Department of Medical Oncology Sir Charles Gairdner Hospital Perth WA Australia; ^4^ National Centre for Asbestos Related Diseases University of Western Australia Perth WA Australia; ^5^ Centre for Respiratory Health, School of Medicine University of Western Australia Perth WA Australia; ^6^ Department of Medicine University of Hong Kong Hong Kong

**Keywords:** Mesothelioma, pleural effusion, pulmonary infiltrate, video‐assisted thoracoscopy

## Abstract

We report a rare case of early and extensive pulmonary invasion of malignant pleural mesothelioma (MPM) in a 70‐year‐old woman. She first presented with a hydropneumothorax and subsequent workup, including video‐assisted thoracoscopy (VAT), confirmed MPM. After VAT, she developed dyspnoea, cough, and widespread pulmonary infiltrates of uncertain aetiology. These infiltrates progressed over the following months, failed to respond to antibiotics, and were strongly fluorodeoxyglucose (FDG)‐avid on positron emission tomography (PET). Bronchoalveolar lavage (BAL) yielded extremely viscous fluid containing mesothelioma cells. These cells were also found in the sputum when nebulized deoxyribonuclease (DNase) was trialled to enhance clearance of the pulmonary fluid. The patient deteriorated rapidly with progressive mediastinal and contralateral MPM involvement and died one month later. This case highlights the importance of including tumour invasion as a differential diagnosis of non‐resolving pulmonary infiltrates in patients with MPM.

## Graphical abstract

We report a rare case of early and extensive pulmonary invasion of malignant pleural mesothelioma (MPM) first presenting with hydropneumothorax. She developed widespread and rapidly progressive FDG‐avid pulmonary infiltrates. Bronchoalveolar lavage (BAL) and sputum contained mesothelioma cells. The patient deteriorated rapidly with progressive mediastinal and contralateral MPM involvement and died one month later. This case highlights the importance of including tumour invasion as a differential diagnosis of non‐resolving pulmonary infiltrates in patients with MPM.

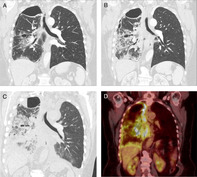

## Introduction

Malignant pleural mesothelioma (MPM) is a neoplasm arising from pleural mesothelial cells and is usually confined within the pleural cavity until later stages of disease [1]. When patients with MPM develop pulmonary infiltrates, the most common differential diagnoses include infection, lymphangitis, or drug reaction (e.g. immunotherapy‐related pneumonitis). Only one case report has described direct invasion of mesothelioma cells into the lung parenchyma (and at a late stage) [2], the mechanism for which was unclear. Large autopsy case series have found a high rate of intraparenchymal mesothelioma invasion [3, 4]. The pattern of this growth was seen to closely resemble other neoplastic and reactive/inflammatory lung diseases, and the authors warn of the risk of misdiagnosis [3].

We report a patient with MPM with pulmonary infiltrates early in her presentation and causing diagnostic uncertainty. Bronchoscopic lavage and biopsies confirmed mesothelioma cells, which were subsequently also found in sputum.

## Case Report

A previously well 70‐year‐old woman presented with exertional dyspnoea. She had domestic exposure to asbestos in her teenage years and a distant past history of non‐Hodgkin's lymphoma. Computed tomography (CT) uncovered a right hydropneumothorax that required chest tube drainage, but no lymphadenopathy or asbestosis. The pleural fluid cytology was suspicious for mesothelioma. Chest radiograph performed the day after tube removal showed evidence of fluid recurrence and a small hydropneumothorax.

The patient underwent video‐assisted thoracoscopy (VAT) with right lung and pleural biopsy (which confirmed epithelioid mesothelioma) and talc poudrage. The post‐operative course was complicated by prolonged air leak necessitating chest tube drainage for two weeks. A repeat CT chest post‐VAT showed re‐accumulation of the effusion but minimal pleural rind and no thoracic lymphadenopathy.

**Figure 1:**
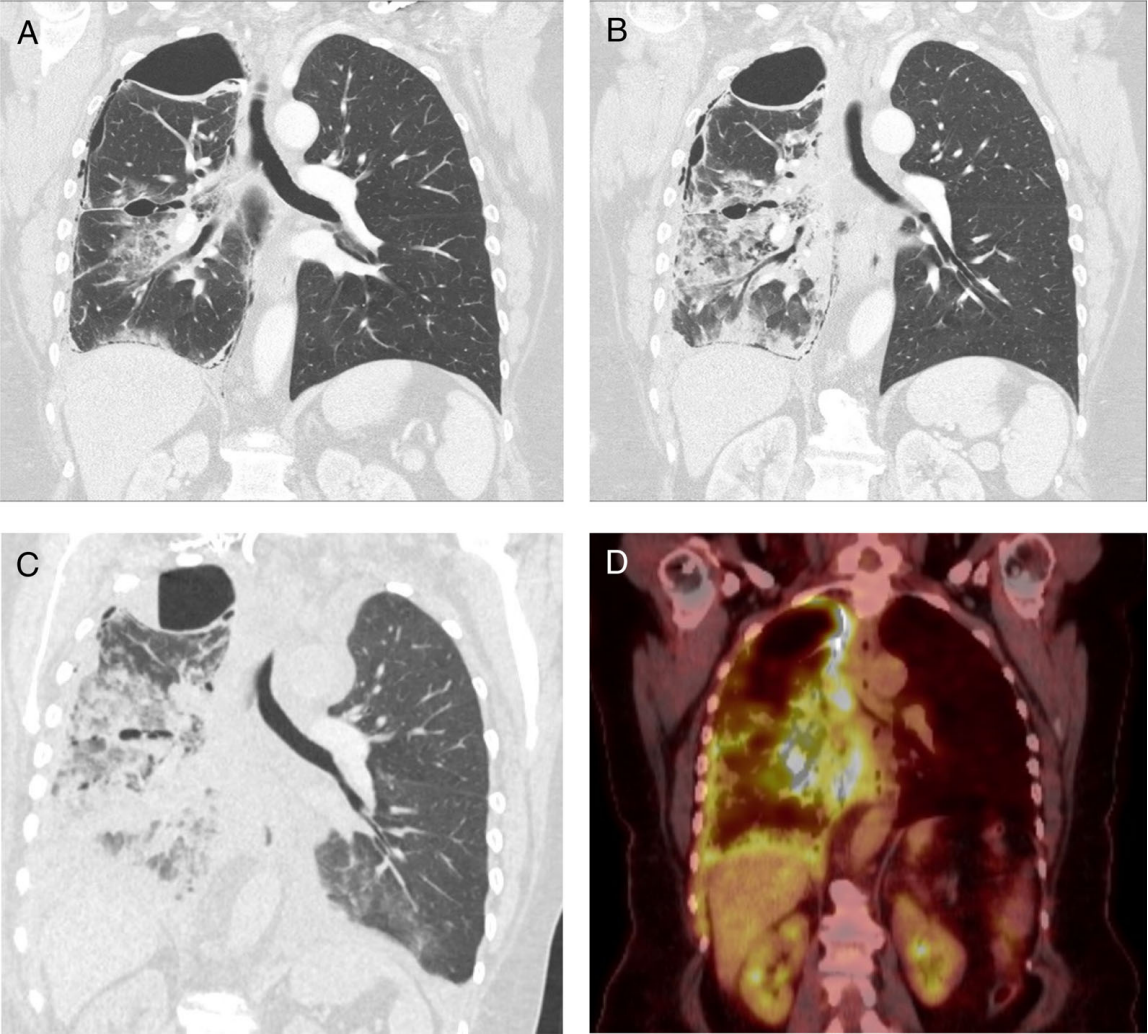
Evolution of pulmonary infiltrates in the right middle and lower lobes at six weeks (A), four months (B), and five months post video‐assisted thoracoscopy (VAT) biopsy (C). Positron emission tomography (PET) demonstrates these infiltrates are FDG‐avid (D).

Palliative chemotherapy was not initiated at that point because of minimal pleural disease and relatively mild symptoms. A surveillance CT was performed six weeks post‐VAT biopsy that showed mild increase in the size of pleural nodules and new, non‐specific ground‐glass changes within the right middle and lower lobes (Fig. 1A). She had not received any new medications during that time. She had a cough but no other infective symptoms or peripheral blood leucocytosis. A trial of antibiotics was commenced without benefits. Her cough, dyspnoea, and exercise tolerance worsened, and she reported a right‐sided chest pain. At the time of referral for consideration of systemic therapy (four months post‐VAT biopsy), a repeat CT confirmed significant progression of her pulmonary infiltrates (Fig. 1B) that were FDG positron emission tomography (PET)‐avid (Fig. 1D), as well as her pleural mesothelioma and intrathoracic lymphadenopathy. Her total leucocyte count was 6.4 × 10^9^/L and C‐reactive protein (CRP) was 11 IU/L. Atypical infection, lymphangitis carcinomatosis, and tumour infiltration were considered. A semi‐urgent bronchoscopy was performed.

**Figure 2:**
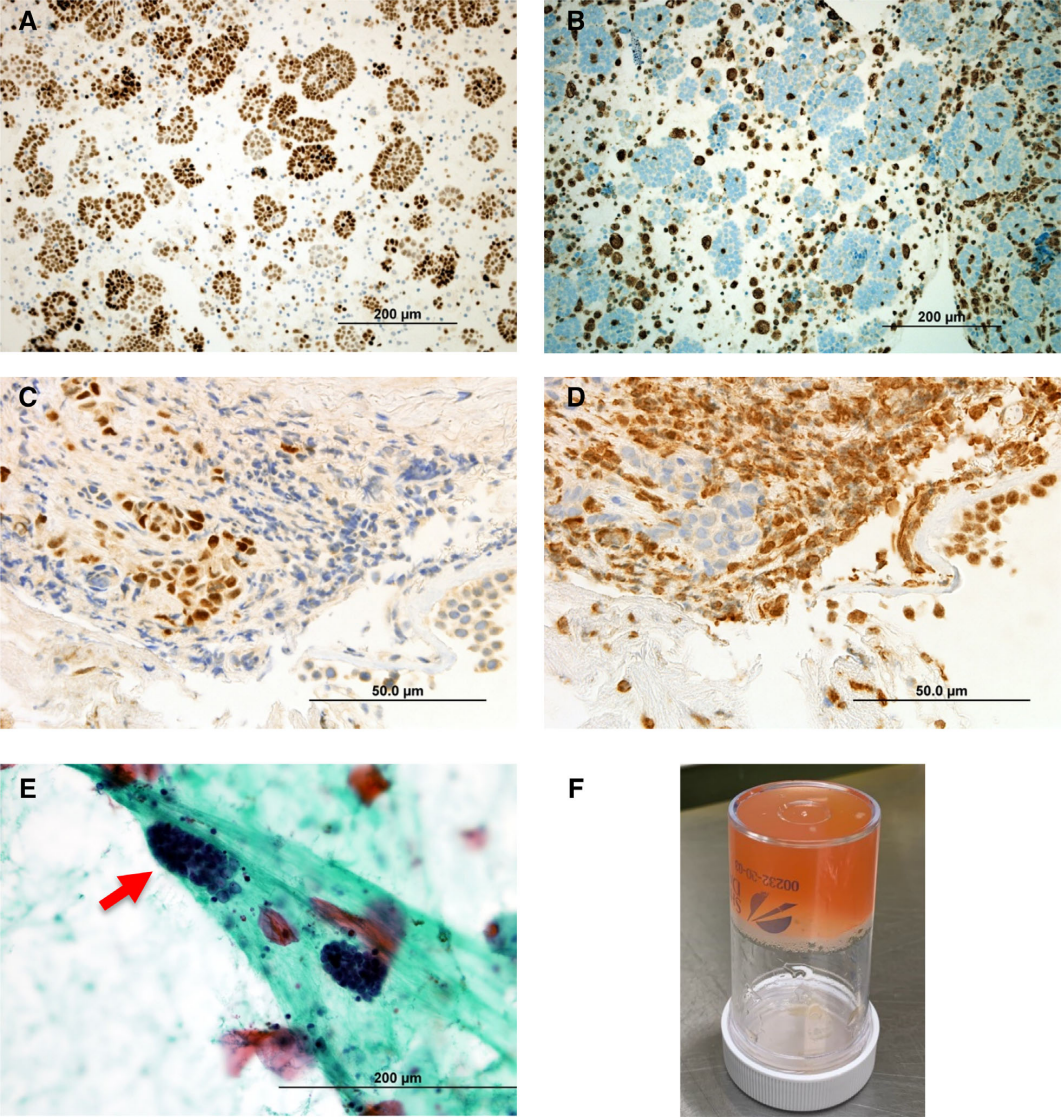
Bronchoalveolar lavage (BAL) cell block (A, B) and bronchial biopsy (C, D) specimens with 3,3′‐diaminobenzidine (DAB) immunohistochemistry (IHC). DAB IHC against Wilms tumour 1 (WT‐1) protein demonstrates positive nuclear (brown) staining in tumour cells present in the BAL cell block (A) and bronchial biopsy (C). DAB IHC against BRCA1‐associated protein‐1 (BAP‐1) in the BAL cell block (B) and biopsy (D) demonstrates loss of expression in tumour cells, supporting the diagnosis of malignant mesothelioma. Papanicolaou‐stained sputum (E) shows the presence of clusters of malignant mesothelioma cells (red arrow). The high viscosity of BAL liquid is displayed in the clotted sample (F).

Bronchoalveolar lavage (BAL), bronchial biopsy, and transbronchial needle aspiration of hilar lymph nodes and lung were positive for malignant mesothelioma, and without evidence of infection (Fig. 2A–C). The lavage fluid was exceedingly viscous and clotted on standing (Fig. 2E). Immediately post‐bronchoscopy, the patient was empirically covered with systemic antibiotics and steroids, but without benefits.

The patient's cough became almost constant and distressing, presumably from the viscous endobronchial secretions. A trial of nebulized deoxyribonuclease (DNase) was initiated to enhance airway clearance. The sputum expectorated contained mesothelioma cells (Fig. 2D).

The patient deteriorated rapidly. Four days post‐bronchoscopy, she developed a new contralateral pleural effusion, and 900 mL of (biochemically confirmed) chylous fluid was drained. A pericardial effusion (without tamponade) was confirmed on echocardiogram. CT showed further progression of her right lung infiltrates (Fig. 1C). Her poor performance status [Eastern Cooperative Oncology Group (ECOG) 4] failed to improve and she was transferred to the hospice for end‐of‐life care and died five weeks later.

## Discussion

Direct and extensive MPM infiltration into the lung parenchyma is rare. Pulmonary infiltrates in the context of thoracic cancer are most commonly caused by infection, lymphangitis carcinomatosis, or drug‐related pneumonitis. Our patient developed diffuse non‐specific pulmonary infiltrates early in the disease course, associated with significant progressive dyspnoea and troublesome cough. When pulmonary mesothelioma invasion was diagnosed, her ECOG performance status had declined rapidly from 1 to 4 and she was not suitable for any systemic therapy. This case suggests that, although rare, mesothelioma invasion should be included as a differential diagnosis of non‐specific pulmonary infiltrates.

How mesothelioma cells invaded into the lung is unknown. Our patient had a malignant pleural effusion and pneumothorax at presentation suggestive of a bronchopulmonary communication. It is possible that mesothelioma cells in the pleural fluid could have migrated into the lung through a bronchopulmonary fistula. She had a prolonged post‐VAT air leak that could have furthered this risk, as the pulmonary infiltrates were only detected on CT after surgery.

Pulmonary invasion of MPM has seldom been described. Chahinian et al. [2] reported one other mesothelioma patient who underwent thoracotomy and partial parietal pleurectomy. The patient developed mesothelioma infiltration of the lung, albeit after two years—a much longer time post‐operatively than our patient, which was confirmed on open lung biopsy that showed replacement of alveolar cells with mesothelioma. Small numbers of histopathological cases of intraparenchymal MPM describe a diversity of growth patterns that mimic other conditions including bronchoalveolar carcinoma and interstitial pneumonia [3].

In our patient, the presence of mesothelioma contributed to thick endobronchial secretions that likely contributed to the distressing cough. A trial of DNase enhanced expectoration of the secretion that showed mesothelioma cells, a rare finding in sputum samples. Unfortunately, the DNase was not sufficiently effective to produce a clinical benefit.

This case describes the rare occurrence of pulmonary MPM invasion as the cause of non‐specific pulmonary infiltrates, and the resultant diagnostic uncertainty. Non‐resolving, or progressive, pulmonary infiltrates therapy in the setting of MPM should be aggressively investigated. Early use of bronchoscopic biopsy can help clarify diagnosis. Clinicians should include mesothelioma invasion of the lung parenchyma as a differential diagnosis of pulmonary infiltrates even in early disease stage.

### Disclosure Statements

Appropriate written informed consent was obtained for publication of this case report and accompanying images.

Y. C. Gary Lee is a recipient of a Medical Research Future Fund Practitioner Fellowship. R. Thomas is a recipient of a National Health & Medical Research Council Early Career Fellowship.
